# Synthesis of 2MP-CuNPs Fluorescent Probes and Their Application in Tetracycline Detection

**DOI:** 10.3390/s24227325

**Published:** 2024-11-16

**Authors:** Qiaoya Dou, Zulpiye Hasanjan, Hongyan Zhang

**Affiliations:** 1 School of Physical Science and Technology, Xinjiang University, Urumqi 830017, China; douqiaoya@xju.edu.cn (Q.D.); zuhjxj@163.com (Z.H.); 2 Xinjiang Key Laboratory of Solid State Physics and Devices, Xinjiang University, Urumqi 830017, China

**Keywords:** 2-mercaptopyridine-copper nanoparticles, fluorescent probe, tetracycline, food inspection

## Abstract

A fluorescent probe composed of 2-mercaptopyridine–copper nanoparticles (2MP-CuNPs) was synthesized through a hydrothermal method utilizing CuCl_2_ and 2-mercaptopyridine (2MP). The experimental results indicate that the 2MP-CuNPs probe exhibited an excellent fluorescence emission peak at 525 nm with an excitation wavelength of 200 nm. Furthermore, this emission peak was accompanied by a substantial Stokes shift of 325 nm, which effectively minimized the overlap between the excitation and emission spectra, thereby enhancing detection sensitivity. In tetracycline (TC) detection, the dimethylamino group on TC undergoes protonation in acidic conditions, resulting in a H^+^ ion. Consequently, the nitrogen atom within the pyridine moiety of the 2MP-CuNPs probe forms a coordination complex with H^+^ via multi-toothed n-bonding interactions, leading to a significant reduction in fluorescence intensity at 525 nm. Based on this mechanism, a quantitative detection method for TC was successfully established with a linear range spanning from 0.1 to 240 µM and an impressive detection limit of 120 nM. Furthermore, during actual sample analyses involving milk and chicken feed, this analysis method based on the 2MP-CuNPs probe achieved absolute recovery rates ranging from 94% to 98%, underscoring its considerable potential for practical applications.

## 1. Introduction

Tetracycline (TC) is a broad-spectrum antibiotic characterized by a hydrogenated tetraphenyl ring as its core structure. This drug has been utilized for over 70 years, exerting its antibacterial effects by specifically binding to the 30 s subunit of bacterial ribosomes, which inhibits peptide chain elongation and disrupts protein synthesis [1]. Notably, TC is affordable, easy to use, and widely employed in veterinary medicine. However, the growth of livestock farming has led to issues such as overfeeding and inadequate isolation space, resulting in frequent disease outbreaks among farm animals [2]. To combat these epidemics and promote the growth of livestock and aquatic species, farms commonly supplement feed with antibiotics, leading to excessive antibiotic consumption and the presence of tetracycline residues in various animal-derived foods [3]. Consequently, there is an urgent need for a rapid and straightforward method to detect TC content in real samples.

To improve the performance of the fluorescent probe in terms of sensitivity and quantification, 2-mercaptopyridine (2MP) was chosen as an appropriate ligand for constructing a copper nanoparticle-based probe for TC detection in this study. Upon coordination, the lone pairs of electrons on the nitrogen (N) and sulfur (S) atoms in (2MP) form stable Cu-S bonds with Cu ions. This interaction induces the splitting of the 3 d^10^ energy levels near the Cu Fermi energy, increases molecular rigidity, and facilitates electron transfer between the ligand and copper, significantly boosting the fluorescence quantum yield [4]. Furthermore, the MP molecule exhibits rotational freedom between the sulfhydryl group and the pyridine ring. During excitation, energy is transferred within the molecule through various mechanisms, such as vibration and rotation, resulting in a substantial Stokes shift that makes the emission wavelength longer than the absorption wavelength. The Stokes shift, a key photophysical parameter of fluorescent probes, indicates the energy loss between the absorption and emission wavelengths. A larger Stokes shift not only minimizes the interference from background excitation light but also mitigates self-burst phenomena, enhancing the probe’s sensitivity [5]. Incorporating 2MP shifts the detection conditions from alkaline to acidic, further improving the comprehensive and accurate detection of TC.

In this study, an effective fluorescent probe using 2-mercaptopyridine–copper nanoparticles (2MP-CuNPs) was synthesized by 2-mercaptopyridine (2MP) and copper chloride (CuCl_2_), and exhibited strong fluorescence emission at 525 nm with a significant Stokes shift of 325 nm when excited at 200 nm. Furthermore, this 2MP-CuNPs probe demonstrated a good fluorescence response to TC. In acidic conditions, the protonated dimethylamine of TC coordinated with the N in the pyridine ring of the 2MP-CuNPs via a polydentate n-bond to form a non-fluorescent complex that diminished the fluorescence intensity. The fluorescence intensity of the 2MP-CuNPs was linearly related to TC concentration within the range of 0–240 µM, achieving a limit of detection (LOD) as low as 120 nM. Moreover, the practical use of the 2MP-CuNPs probe was confirmed through the detection of TC residues in samples of milk and chicken feed.

## 2. Materials and Methods

### 2.1. Material and Reagents

TC was acquired from Bidepharm (Shanghai, China), while sodium hydroxide (NaOH), CuCl_2_, iron chloride (FeCl_3_), glucose (Glu), ascorbic acid (AA), and sodium chloride (NaCl) were sourced from Sangon Biotech (Shanghai, China). Glycine (Gly), lysine (Lys), valine (Val), isoleucine (Iso), 2MP, methionine (Met), and calcium chloride (CaCl_2_) were obtained from Macklin (Shanghai, China). Lithium chloride (LiCl), polyvinylpyrrolidone (PVP), zinc chloride (ZnCl_2_), magnesium chloride (MgCl_2_), potassium hydroxide (KOH), ethyl acetate, n-hexane, and sodium sulfate (Na_2_SO_4_) were sourced from Aladdin Reagent Co., Ltd (Shanghai, China). All the chemicals used were of analytical grade (AR), and deionized water from a pure water system was employed in the experiments.

### 2.2. Materials Characterization

The structure of the Cu nanoparticles was analyzed using a Bruker D8 Advance X-ray diffraction system (XRD, Bruker, Karlsruhe, Germany). The morphology, size, and lattice structure of the copper nanoparticles were examined using a JEM-2100F transmission electron microscope (TEM, JEM-2100F, Hitachi, Tokyo, Japan). Additionally, absorption spectra were recorded at room temperature (25 °C) using a Lambda 650 UV–Vis spectrophotometer (UV–Vis Lambda 65, PerkinElmer, Waltham, MA, USA). Meanwhile, a Fourier transform infrared spectrometer (FT-IR; VERTEX 70, Bruker, Germany) was employed to analyze the functional groups of the samples. Fluorescence measurements were taken using an F-4600 fluorescence spectrometer (Hitachi, Japan) with an operating voltage of 660 V and excitation/emission wavelengths of 10.0/10.0 nm, respectively.

### 2.3. Synthesis of Fluorescent Probes for 2MP-CuNPs

**Figure 1:**
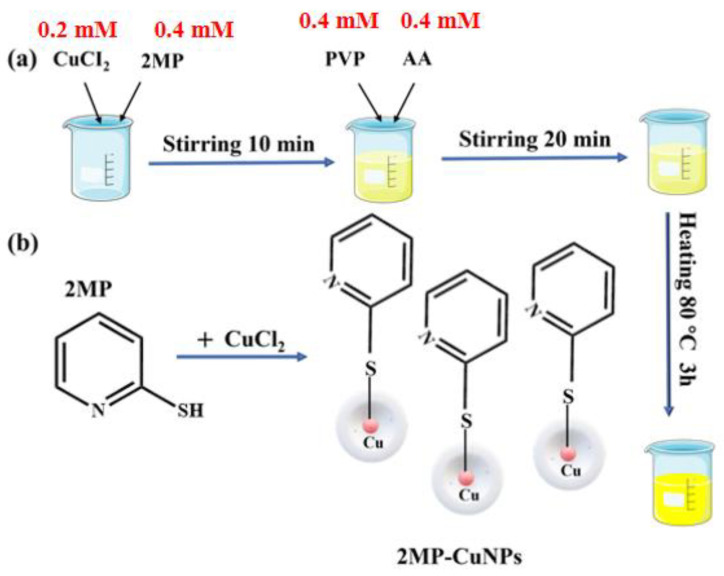
(**a**) Synthesis process of 2MP-CuNPs probe; (**b**) 2MP-CuNPs probe reaction process.

First, 26.8 mg of CuCl_2_ (0.2 mM) and 45 mg of 2MP (0.4 mM) were dissolved in 200 mL of deionized water and stirred for 10 min, resulting in a pale yellow solution. Next, 70 mg of AA (0.4 mM) and 45 mg of PVP (0.4 mM) were added to this solution, and stirring continued for another 20 min to ensure thorough mixing. Finally, the mixture was reacted at 80 °C for 3 h to yield dark yellow 2MP-CuNPs as fluorescent probes. The resulting solution was stored at 4 °C for later use. The preparation and reaction process for the 2MP-CuNPs probes are illustrated in Figure 1a and Figure 1b, respectively.

### 2.4. Fluorescence Detection of TC

At room temperature (RT, 25 °C), 1 mL of the 2MP-CuNPs probe solution and 1 mL of PBS buffer solution at pH 6.0 were each transferred into separate 5 mL colorimetric tubes and incubated for 3 min to ensure thorough mixing. Next, varying concentrations of TC solution were added to the mixture, and the reaction was allowed to proceed for another 3 min at RT. Finally, the solution was excited at an excitation wavelength of 200 nm, and the resulting fluorescence emission data were recorded. To investigate the selective detection of TC by the 2MP-CuNPs probe, various potential interferences were introduced into the probe and PBS buffer solution, including Ca^2+^, Na^+^, K^+^, Li^+^, Mg^2+^, Zn^2+^, Fe^3+^, Cl^−^, OH^−^, SO_4_^2−^, Met, Gly, Iso, Val, Lys, and Glu. The reaction occurred at room temperature for 3 min, and the fluorescence spectra were recorded under consistent conditions. For competitive detection of TC using the 2MP-CuNPs, coexisting solutions of TC and various interferences were prepared in PBS. The same list of interferences was used, and each coexisting solution was added to the 2MP-CuNPs probes, followed by a 3 min reaction at room temperature, with the fluorescence spectra recorded under identical conditions.

### 2.5. TC Detection in Real Samples

To assess the reliability of the 2MP-CuNPs probe for detecting TC in real samples, chicken feed and milk were chosen for residue analysis. The samples’ preparation followed established protocols [6]. Initially, 6 g of commercially available chicken feed were placed in a centrifuge tube with 15 mL of ethyl acetate and stirred for 1 min. Following this, ultrasonic extraction was conducted for 20 min, after which the supernatant was collected and centrifuged at 5000 rpm for 15 min. The residue was then re-extracted with an additional 10 mL of ethyl acetate and combined with the supernatant. To dissolve the residue, 10 mL of 4% NaCl solution and 10 mL of n-hexane were added sequentially; after allowing the mixture to stand for 2 min to separate, the upper n-hexane layer was discarded and filtered through a 0.22 µM membrane. To reduce interference during fluorescence detection, 3 mL of milk samples were diluted with 6 mL of ultrapure water. Acknowledging that the proteins and fats in milk could impact the TC analysis, we added 10% trichloroacetic acid and 2 mL of trichloromethane to the diluted milk in a centrifuge tube, vortexed for 90 s, and then sonicated for 20 min. The samples were centrifuged twice at 10,000 rpm for 15 min each, and the resulting supernatant was utilized for TC assays. Finally, various concentrations of TC solutions were mixed with the pre-treated chicken feed and milk samples, and the fluorescence spectra of these mixtures were measured to evaluate the performance of the 2MP-CuNPs probe in determining TC in real samples.

## 3. Results

### 3.1. Characterization of 2MP-CuNPs Fluorescent Probes

**Figure 2:**
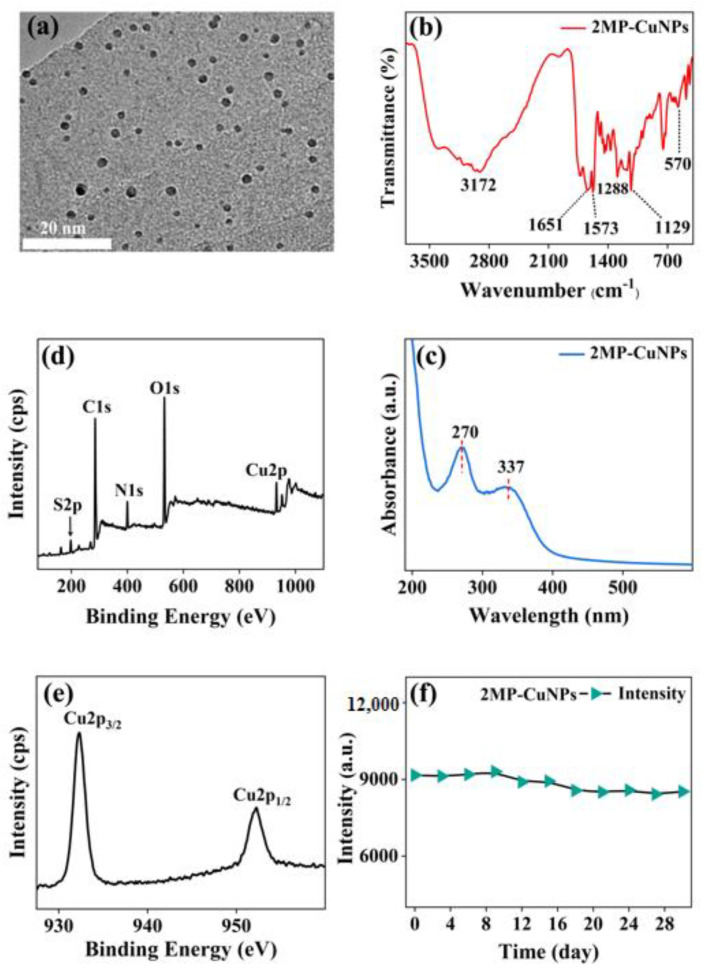
(**a**) TEM, (**b**) infrared spectrum, (**c**) UV-Vis absorption spectrum, (**d**) XPS spectrum, (**e**) XPS spectrum of Cu 2p, and (**f**) stability diagram of 2MP-CuNPs probe.

The morphology and structure of the 2MP-CuNPs probes were analyzed using transmission electron microscopy. In Figure 2a, the probes exhibit a nanosphere shape with a diameter of approximately 3 nm and demonstrate excellent dispersion. This dispersibility is attributed to the hydrophobic carbon chains in PVP, which effectively prevent nanoparticle aggregation through strong repulsive forces in the aqueous phase, thereby stabilizing the reaction system [7]. To investigate the surface functional groups of the 2MP-CuNPs probes, Fourier transform infrared (FTIR) spectroscopy was utilized. In Figure 2b, the absorption peak at 3172 cm^−1^ corresponds to the vibration of the hydroxyl group (-OH), indicating that the 2MP-CuNPs probe has good water solubility. The peaks at 1573 cm^−1^ and 1651 cm^−1^ are attributed to the stretching vibration of the N-H bond and the bending vibration of the C=O bond [8]. Furthermore, the peaks at 1129 cm^−1^ and 1288 cm^−1^ correspond to the stretching vibration of the C-O-C bond and the symmetric stretching of the C-O bond [9]. The absorption peak at 570 cm^−1^ signifies the stretching vibration of the Cu-S bond, indicating the formation of a Cu-S bond between 2MP and Cu [10]. Figure 2c illustrates the ultraviolet–visible absorption spectrum of the 2MP-CuNPs probe. The absorption peak observed at 270 nm for the 2MP-CuNPs probe is attributed to the transition absorption involving the lone electron pairs of N and S atoms in the p orbitals of the 2MP ligand, as well as the extensive π bond character associated with the benzene ring. Meanwhile, the absorption peak at 337 nm corresponds to the n→π* transition arising from copper(II) interacting with 2MP [11]. An X-ray photoelectron spectroscopy (XPS) analysis of the 2MP-CuNPs probe was conducted. Figure 2d reveals five peaks at 198.83 eV, 286.95 eV, 399.76 eV, 530.93 eV, and 931.86 eV, corresponding to S2p, C1s, N1s, O1s, and Cu2p, respectively [12]. The S, C, and N signals originate from 2MP, Cu comes from CuCl₂, and O is derived from PVP and ascorbic acid.

**Figure 3:**
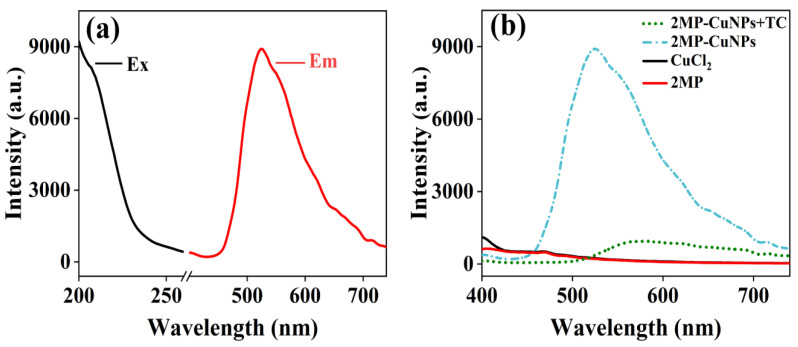
(**a**) Ex/Em spectra of the 2MP-CuNPs probe; (**b**) fluorescence spectra of 2MP, CuCl_2_, 2MP-CuNPs, and 2MP-CuNPs+TC.

In Figure 2e, the Cu spectra exhibit two absorption peaks at 931.74 eV and 951.97 eV, corresponding to Cu 2p_3/2_ and Cu 2p_1/2_, respectively. This indicates that the CuNPs in the 2MP-CuNPs probe consist of both Cu(0) and Cu(I) components [13]. Additionally, the absence of a peak at 942.0 eV confirms that there is no Cu^2+^ residue in the 2MP-CuNPs probe. The stability of the 2MP-CuNPs probe was assessed. In Figure 2f, the fluorescence intensity remained largely stable after 28 days of storage, demonstrating its good stability and suitability for practical applications. In Figure 3a, under 200 nm excitation, the 2MP-CuNPs probe has a maximum emission wavelength of 525 nm. The probe exhibits a large Stokes shift of 325 nm, which minimizes the self-quenching of fluorescence and enhances both the sensitivity and accuracy in detection [14,15]. The fluorescence spectra were also recorded for the CuCl₂ solution, 2MP solution, 2MP-CuNPs probe, and 2MP-CuNPs probe after reaction with 240 mM TC, all under 200 nm excitation. In Figure 3b, it can be seen that the CuCl₂ and 2MP solutions displayed weak fluorescence emission at 400 nm, whereas the 2MP-CuNPs probe emitted strong fluorescence at 525 nm. This enhanced fluorescence is attributed to the charge transfer interaction between 2MP and Cu within the 2MP-CuNPs probe, which increases its fluorescence quantum yield. Furthermore, the fluorescence intensity of the 2MP-CuNPs probe decreased significantly upon reaction with 240 μM TC, indicating its potential for sensitive and rapid TC detection.

### 3.2. Optimization of 2MP-CuNPs Fluorescent Probes

**Figure 4:**
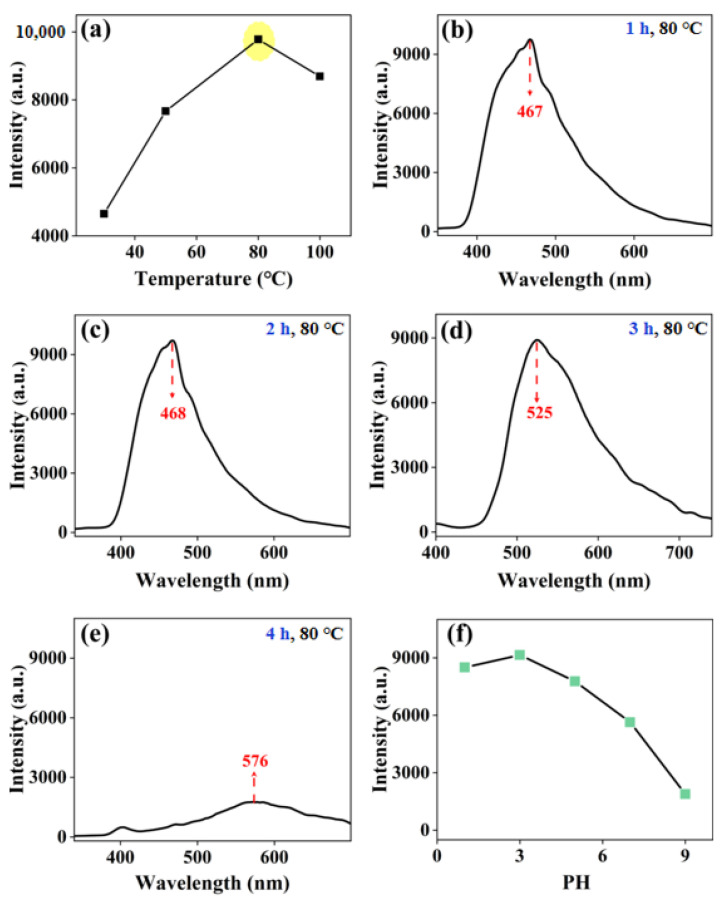
(**a**) Optimization of the reaction temperature for 2MP-CuNPs probes; (**b**–**e**) optimization of the reaction time for 2MP-CuNPs probes; (**f**) influence of pH on the fluorescence intensity of 2MP-CuNPs probes.

To optimize the synthesis conditions, first, the effect of the synthesis temperature on the fluorescence intensity of the 2MP-CuNPs probe was studied. The solution was heated in a water bath for 1 h at temperatures of 30 °C, 50 °C, 80 °C, and 100 °C, followed by recording the fluorescence spectrum of the 2MP-CuNPs probe. In Figure 4a, it can be seen that the fluorescence intensity of the 2MP-CuNPs probe increased steadily from 30 to 80 °C. However, beyond 80 °C, the intensity began to decrease. This decline is attributed to the pyrolysis of 2MP near 100 °C, which causes its decomposition products to detach from the copper nanoparticles, destabilizing the surface modification layer and reducing the fluorescence intensity [16]. Therefore, 80 °C is the optimal synthesis temperature.

Next, the synthesis temperature of the 2MP-CuNPs probe was fixed at 80 °C, and the influence of different synthesis times on the fluorescence intensity was studied. In Figure 4b–e, when the synthesis time is 1 h, 2 h, and 3 h, the changes in fluorescence intensity are not significant, but the position of the fluorescence peak is altered. At 1 h and 2 h, the fluorescence peaks were located at 467 nm and 468 nm, respectively, while at 3 h, the fluorescence peak of the 2MP-CuNPs probe shifted to 525 nm. This is attributed to a lower quantity of Cu nanoparticles and a thinner modification layer at shorter synthesis times. As synthesis continued, particularly at 3 h, both the number of Cu nanoparticles and the thickness of the modification layer increased, causing the peak to shift to 525 nm. When the synthesis time extended to 4 h, the peak further shifted to 576 nm, but the fluorescence intensity significantly decreased. Therefore, the synthesis was carried out by water-bath heating at 80 °C for 3 h, at which time the Stokes shift of the 2MP-CuNPs probe was at its maximum, which could reduce the self-quenching of the probe’s fluorescence and improve the sensitivity and accuracy of detection [17].

Finally, the synthesis temperature and time for fixing the 2MP-CuNPs probe were set at 80 °C and 3 h, respectively. The influence of different pH environments on the fluorescence intensity of the probe was studied. In Figure 4f, the 2MP-CuNPs probe exhibited the highest fluorescence intensity at pH = 3. This may be because in acidic conditions, the thiol functional group will negatively ionize to form thiolate anions, which can bind to Cu ions [5,18]. This binding can effectively limit the non-radiative recombination process on the surface of Cu nanoparticles, thereby enhancing the fluorescence intensity. Therefore, we ultimately chose pH = 3 and 80 °C water-bath heating for 3 h as the optimal synthesis conditions for the 2MP-CuNPs probe and used it for TC detection.

### 3.3. Selectivity and Sensitivity of 2MP-CuNPs Probes in Detecting TC

**Figure 5:**
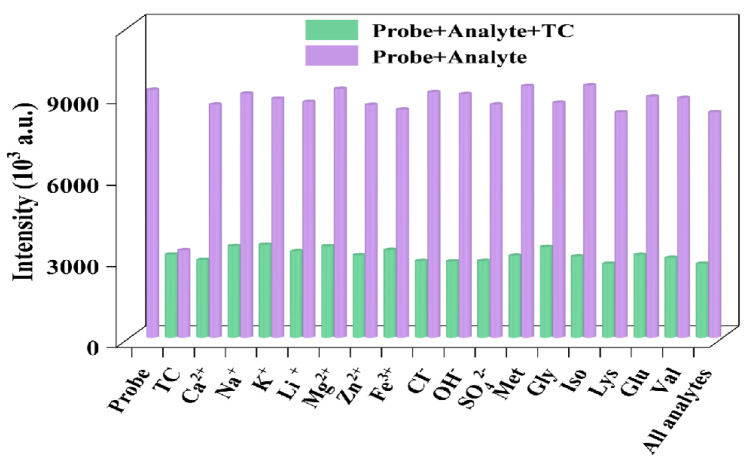
The impact of various interferents at a concentration of 0.2 mM on the fluorescence intensity of the 2MP-CuNPs probe was assessed, including scenarios where 0.2 mM tetracycline (TC) is present and absent, with all the analytes and interferents being evaluated simultaneously.

To assess the selectivity of the 2MP-CuNPs probe for TC detection, the response of the 2MP-CuNPs probes to different interferents was studied. The mixture contained various interferents, including Ca^2+^, Na⁺, K⁺, Li⁺, Mg^2+^, Zn^2+^, and Fe^3+^, as well as anions, such as Cl^−^, OH^−^, and SO_4_^2−^. The amino acids present were methionine, glucose, glycine, isoleucine, valine, lysine, and TC. All the interferents and TC were maintained at a concentration of 0.2 mM. As can be seen in Figure 5, with the exception of TC, the other interferents did not influence the fluorescence intensity of the 2MP-CuNPs probe, thereby demonstrating that the 2MP-CuNPs probe exhibits excellent selectivity for TC detection. Furthermore, the influence of multiple interfering substances on the fluorescence intensity of the 2MP-CuNPs probe when they are co-present has also been explored. The findings demonstrate that the fluorescence intensity alteration of the 2MP-CuNPs probe was insignificant. This could be accounted for by the fact that the 2MP-CuNPs probe exhibits excellent accuracy in TC detection. To appraise the immunity to interference of the 2MP-CuNPs probe, supplementary investigations were executed to scrutinize the fluorescence response of the 2MP-CuNPs probe at a concentration of 0.2 mM in the presence of multiple interferents at a concentration of 0.2 mM TC. In Figure 5, it is evident that the interaction between the 2MP-CuNPs probe and TC remained unperturbed by other constituents, even in the concurrent presence of multiple interferents. These substances did not impede the interaction between the 2MP-CuNPs probe and TC, and TC could still conspicuously attenuate the fluorescence intensity of the 2MP-CuNPs probe. These outcomes substantiate that the 2MP-CuNPs probe possesses outstanding anti-interference capability in the fluorescence detection of TC.

**Figure 6:**
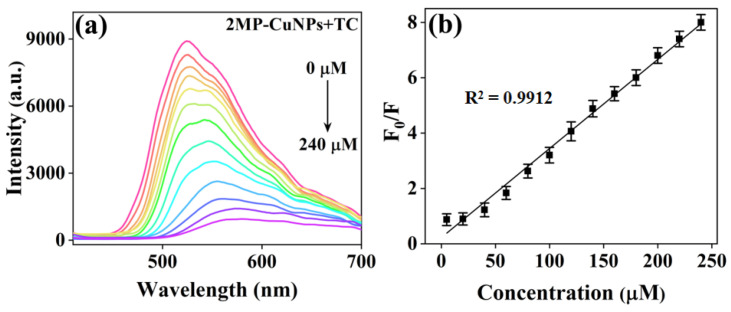
(**a**) Fluorescence spectra of 2MP-CuNPs probes at tetracycline (TC) concentrations ranging from 0 to 240 µM (λex = 200 nm, lem = 500 nm); (**b**) linear plot showing the relationship between the relative fluorescence intensity (F₀/F) of 2MP-CuNPs probes and TC concentration within the 5 to 240 µM range.

The concentration of TC was measured under optimal synthesis conditions. In Figure 6a, upon the addition of varying concentrations of TC (0, 5, 20, 40, 60, 80, 100, 120, 140, 160, 180, 200, and 240 μM) to the probe composed of 2MP-CuNPs, a gradual decrease in fluorescence intensity is observed as the concentration of TC increases. Once the concentration surpassed 240 μM, the fluorescence intensity quenching of the 2MP-CuNPs probe reached a saturation point and ceased to decline further. Additionally, it was noted that the fluorescence peak position of the 2MP-CuNPs probe is influenced by TC, with a shift in the fluorescence peak from 525 nm to 565 nm. This phenomenon occurs due to the nucleophilic attack of TC on the surface of CuNPs, which increases the coordination distance between 2MP and Cu, resulting in a modification of the energy difference between the excited state and ground state of the probe molecule, thereby affecting its fluorescence emission peak position [19]. In Figure 6b, the relative fluorescence intensity (F0/F) exhibits a strong linear relationship with the TC concentration (Q) within the range of 0–240 μM; the linear regression equation for the detection platform is expressed as F0/F = 0.0322Q-0.2268 (R^2^ = 0.9912). Here, F_0_ and F denote the fluorescence intensities of the 2MP-CuNPs probes prior to and following TC addition, respectively. Utilizing the formula for calculating the limit of detection (LOD = 3 δ/k, where δ is the standard deviation and k is the slope of the linear curve), the 2MP-CuNPs probe’s detection limit was calculated to be 120 nM. In Table 1, the 2MP-CuNPs probe demonstrates a more pronounced Stokes shift, a lower detection limit, and an extended linear range compared to other fluorescent probes. This suggests that the 2MP-CuNPs probe possesses significant potential and feasibility for detecting TC in real sample matrices.

### 3.4. Detection of TC in Real-World Samples

To assess the feasibility and reproducibility of the 2MP-CuNPs probe in practical applications, it was employed for detecting TC concentrations in milk and chicken feed samples utilizing the standard addition method. Initially, 20 μM and 50 μM TC standards were introduced as standards to the prepared milk and chicken feed samples, respectively. Subsequently, the fluorescence intensity of these samples was systematically measured, leading to the establishment of a linear relationship between the TC detection concentration and fluorescence intensity. In Table 2, the recovery rates for TC addition using the 2MP-CuNPs probe ranged from 94% to 98%, with a relative standard deviation (RSD) below 3.7%. These results demonstrate the high sensitivity and accuracy of the 2MP-CuNPs probe in real samples, thereby showcasing significant potential for application in actual sample assessments.

### 3.5. Analysis of Fluorescence Quenching Mechanism

**Figure 7:**
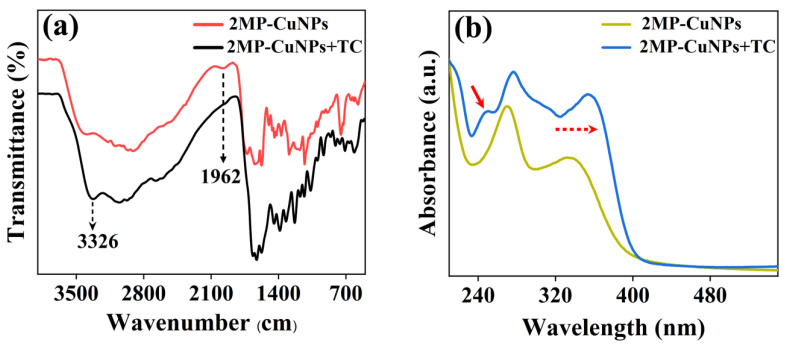
(**a**) Fourier transform infrared spectroscopy of the 2MP-CuNPs probe and the 2MP-CuNPs probe reacting with 80 μM TC; (**b**) UV–Vis spectra of the 2MP-CuNPs probe reacting with 80 μM TC.

The fluorescence quenching mechanism of the 2MP-CuNPs probe in detecting TC was investigated through FTIR and UV–Vis absorption spectroscopy. Figure 7a presents the FTIR spectra of the 2MP-CuNPs probe, as well as those of the 2MP-CuNPs probe upon reaction with 80 μM TC (denoted as 2MP-CuNPs+TC). The results reveal a significant increase in the N-H stretching vibration peak at 3326 cm^−1^ in the 2MP-CuNPs+TC complex, while the C-N bending vibration peak at 1962 cm^−1^ was absent compared to the 2MP-CuNPs probe alone. This alteration may arise from the protonation of the dimethylamine group within the TC molecule under acidic conditions, resulting in a positive charge. The exposed pyridine nitrogen in the 2MP-CuNPs probe interacts with the positively charged TC, resulting in a decrease in fluorescence intensity. The UV–Vis absorption spectra provided further evidence for this phenomenon. In Figure 7b, it can be seen that the introduction of TC caused a redshift and enhancement of the absorption peaks at 270 nm and 337 nm for the 2MP-CuNPs probe, while concurrently generating a new absorption peak at 247 nm. These spectral alterations indicate the formation of novel substances within the system.

## 4. Conclusions

In this study, a fluorescent probe based on 2MP and CuCl_2_ (2MP-CuNPs) was synthesized by a hydrothermal method. The 2MP-CuNPs probe exhibited excellent dispersion in water and emitted strong fluorescence at 525 nm when excited at 200 nm. In acidic conditions, the N atom in the pyridine ring of the 2MP-CuNPs coordinated with the protonated dimethylamine, forming a complex that reduced the fluorescence intensity of 2MP-CuNPs, enabling the detection of the TC concentration. In TC detection, the 2MP-CuNPs probe show a linear range of 0–240 µM and a detection limit of 120 nM for TC. Furthermore, the absolute TC recoveries ranged from 94% to 98% when tested with real milk and chicken feed samples. Thus, the 2MP-CuNPs probe shows great promise for food safety monitoring.

## Figures and Tables

**Table 1:** Comparison of the 2MP-CuNPs probe with other fluorescent probes for TC.

Probe	λex/nm	λem/nm	Stokes Shift nm	LOD (μM)	Linear Range (μM)	Reference
CDs	350	445	95	0.52	2–150	[20]
CuNCs	425	480	55	0.92	3.6–1000	[21]
AgNCs	386	494	108	0.47	1.12–230	[22]
AuNCs-Apt	—	652	—	0.5	1–16	[23]
Paper extract	436	538	102	0.48	1–100	[24]
2MP-CuNPs	200	525	325	0.12	0–240	This work

**Table 2:** Absolute recoveries of TC in milk and chicken feed samples using standardized spiking methods.

Samples	Added (μM)	Measured (μM)	Recovery (%)	RSD (%)
Milk	20.00	18.8	94.00	3.17
	50.00	49.47	98.16	1.63
Chicken feed	20.00	19.46	97.30	3.65
	50.00	48.05	96.10	2.44

## Data Availability

Data are available in a publicly accessible repositor.
